# Transactions of the Medical Society of the County of Kings

**Published:** 1865-07

**Authors:** 


VOL. IV. JULY 1865. No. 12

ART. I.—*Transactions of the Medical Society of the County of Kings*

REGULAR MEETING, APRIL, 1864.

*Epithelioma.*

Dr. Enos presented a specimen of epithelial growth, removed from a patient after death. The subject, a married woman, 58 years of age, had been an office patient of Dr. Kissam, and had been suffering for many years from an ulcer on the middle of the right side of the neck. On the day of her death she had walked a long distance, and on her return homeward hemorrhage occurred in the part affected. Soon afterwards she slipped and fell down an *area*, and when picked up the blood was pouring out in torrents, her garments being completely saturated. On examination the ulcer was found to have involved the *platysma myoides* and *sterno-cleido-mastoid* muscles, producing a degeneration of those tissues. The ulceration had also involved the *internal jugular vein*, which had, probably, given way upon turning her head, before she fell. The fall, most likely, opened the vein more extensively; and the ruptured vessel, remaining open, allowed access to the air, which entered the heart, and produced sudden death. The vessel was surrounded with so much consolidated structure, that the opening could not close up; the sides had been compressed together, adhesive inflammation had taken place, and the vein had, probably, been obliterated. Under the microscope transformed cells were found, and we supposed it to be an epithelial cancer, gradually developing itself in the muscles, and extending through to the skin.

Dr. Minor remarked that he had previously seen the specimen, but had not observed, till now, the existence of a *plug* at the *distal* side of the heart. Had supposed that death was owing to syncope; but the discovery of this *plug* almost demonstrated a different cause for death. In the *diastole* of the heart, air was drawn in through the opening in the vein, and paralysis of the heart supervened. Nature had set up a plastic effusion at the *distal*, and not at the *proximal* side of the heart. An extremely small quantity of air, entering the circulation, will produce fatal results; but he did not think that *asphyxia* would produce instantaneous death.

Dr. Enos supposed the air passed through the pulmonary artery, and thence into the pulmonary capillaries; and these refusing to transmit it, *asphyxia* supervened. No doubt, if the heart is paralyzed, death ensues; but the question arises, "does air, if introduced into the cavity of the heart, produce instant death, by paralysis of that organ?" He thought not, and as a proof, he would refer to a case where he had removed the heart from a serpent, and the heart contracted for half an hour afterwards; hence there was no *paralysis*. When air enters the circulation, it prevents the blood from being transmitted through the pulmonary capillaries; the blood, as a consequence, does not become areated, and death finally ensues.

Dr. Minor replied, that the difference between a reptile and a human heart was so great, that any comparison between them could not be entertained. But why, he would ask, will the ingress of a minute globule of air into the circulation produce instantaneous death? If it were from asphyxia, death would not take place so soon; the comparative slowness of death from this cause would make the explanation untenable. Dr. Dalton says, "no animal will live a *second* after the heart ceases to beat." He (Dr. Minor) thought, therefore, that the air deterred the natural stimulus from the heart, and as a consequence the heart ceases to beat.

Dr. Mason felt very much interested in the discussion, but thought that the cause of death was very obvious. The patient, it seems, was old and feeble, and laboring for a long time under a cancerous disease. On the day of her death she had walked a long distance, was attacked with profuse hemorrhage and died. Setting aside all speculations, he thought it would be sufficient to say, that she died of hemorrhage. With regard to reptiles, the fact is well authenticated, that they will live without breathing, for a long time; perhaps for ages. In the case under consideration, he thought we could not arrive at a different conclusion than that the patient died from sudden exhaustion, the result of excessive hemorrhage.

Dr. Conkling thought that concussion of the brain might have had something to do with the cause of death, as the woman, in falling, struck the forehead in the vicinity of the eye.

*Cerebro-Spinal Meningitis.*

Dr. D. S. Landon reported two cases of cerebro-spinal meningitis. They were characterized by the appearance of rubeola-like spots—more livid towards the last, tetanic spasms and fatality. On post mortem the meninges of the brain and spinal marrow were found to be intensely inflamed.

Dr. Enos saw both of these cases with Dr. Landon, and of the first case particularly, described the sub-arachnoid effusion of ceropurulent matter—limited to the surface of the pia mater, not dipping down into the convolutions. It was not observed around the medulla. The spots were peculiar—gradually passing from center to the circumference, and very profound—that is to say, extending quite through the cutaneous tissue and hemorrhagic.

Dr. Byrne inclines to the belief that the disease in question, is merely a very malignant form of measles, and thinks that upon further investigation the opinion will be confirmed.

Dr. Enos recurred to a case he saw three or four months ago, which, though not characterized by an eruption, he now regarded as *cerebro-spinal meningitis.* The post mortem appearances in that case were very similar to the first case reported by Dr. Landon. The lesion in this case, however, was more particularly at the base of the brain.

Dr. Enos also reported a case of the same disease, in a woman fifty years of age, who was taken with pain in the abdomen and vomiting. She was first seen by a homœopathist, on Wednesday before Easter, who pronounced it to be gastritis. Dr. Enos saw her in the night of the same day, and found her suffering with excessive headache, and general hyperæsthesia. Head thrown back, pupils contracted, eyes suffused, and semi-unconscious.—Surface cold, particularly extremities. Applied leeches to back of neck, but afterwards she appeared to be worse. Opiates only, seemed to give relief. Profuse diaphoresis also supervened under their use. Although she became more tranquil, there was, however, no apparent relief of the disease. The prominent symptoms continued. The urine being retained, on Friday it was drawn off, and found to be albumenous. Saturday a severe herpes labialis appeared, and quickly succeeding this, florid spots about the size of a split pea began to appear on the extremities. On Sunday, Dr. Sands, of New York, was consulted, and coincided in the diagnosis. The patient died on Sunday afternoon. No post mortem could be obtained.

Dr. Enos had also seen another case, in consultation with Dr. Cochran, with similar symptoms in the early stage. He saw her the day before death. The spots were chiefly confined to the extremities, and some of them were from an inch to an inch and a half long by three-quarters of an inch wide, slightly raised, and of a slate color. They appeared to be circumscribed by a definite border, though irregular in shape.

Dr. Mitchell had seen a patient with Dr. Hutchison, in which there was reason to suspect *cerebro-spinal meningitis*, but he had not since heard of it, and could not give the details of the case.

Dr. Bell remarked, that he had seen one of Dr. Landon's cases, which had all the characteristics peculiar to epidemic diseases, where the blood was in a low condition. The *spots* were raised after death, and during the disease, and extended throughout the cutaneous structure. In cutting through them the blood exuded, showing it to be in a fluid condition. This is characteristic of several forms of epidemic diseases of a malignant character.

Dr. Ford thought this disease of great importance. It had appeared in different parts of the country at different periods, but the *spots* were *incidental*, and did not always occur. In 1805 only *one* or *two* in *ten* presented this phenomenon; and at the present time it is not always present. The disease then showed itself by first attacking the feet, and afterwards changing to some other portion of the body. The epidemic, at present, is *adynamic* in character. He had had, he thought, a case of the kind about two months ago, where the patient was first attacked with severe pain in the right elbow; then the right hypochondriac region became involved; the pain was very severe, and pleuritic in character; the whole right side seemed enlarged; the pulse was 80, and very full, and the veins very much distended. Ordered a purge of calomel and compound extract of colocynth, with warm drinks and sinapisms; also administered calomel and opium. The next morning the patient felt better; but in the evening found mucous râles over the right lung, and dulness on percussion. On the morning of the second day he seemed convalescing, and complained of no pain; but during the day was attacked with severe pain in the head. On the third day found him with en intermittent pulse, pain in the back of the neck, and eyes suffused. Ordered a blister to the neck, and administered stimulants, and the patient seemed to improve. A few hours later, however, hemorrhage occurred at the nose, and the patient gradually sank and died.

### *Pneumonia.*

Dr. Mitchell mentioned that he had seen an unusual number of cases of pneumonia, especially in complication with measles, and some of these cases were remarkably rapid. Consolidation, in one case, was evident in thirty-six hours. Another case, complicated with rheumatism, presented a state of complete consolidation after two days duration. There was also pleurisy in this case, at the first; and subsequent to this state of things the pneumonia rapidly recovering, simultaneously with a new attack of rheumatism of the extremities. The disease thus recurred a *third* time consequent upon rheumatic metastasis.

Another case of pneumonia uncomplicated in a previously healthy servant girl, was *relieved in one night* by 20 grains of calomel. Two days afterward she was convalescent.

Dr. Minor traced an apparent prevalence of what appears to be a metastatic predisposition of inflammatory affections. A recent detail of cases of cerebro-spinal meningitis, by Dr. Scriven, of Long Branch, New Jersey, showed that most of his cases of that disease began in rheumatism. These cases of pneumonia by Dr. Mitchell, as an apparent result of rheumatic metastasis, seemed to him to be at least a curious coincidence.

Dr. Byrnes' recent experience was in accord with Dr. Mitchell's. He had had an unusual number of cases of pneumonia, which had generally run a very rapid course, but favorably.

Dr. Ford had also experienced a prevalence of pneumonia, and had noticed the same peculiarities.

### *Ptyalism.*

Dr. Mitchell stated that he had recently noticed what appeared to him to be an extraordinary liability to ptyalism under the use of mercury.

This statement elicited an opinion from Dr. Minor, that the increased susceptibility to mercury in this connection was probably due to the *adynamic* character of diseases. Very different, he thought, from what the same diseases were many years ago, and hence the propriety of the supporting plan of treatment now so generally pursued.

---

### REGULAR MEETING, MAY 17, 1864.

### *Discussion on Rupture of the Uterus.*

The discussion on Rupture of the Uterus being the special order for the evening, Dr. Chapman stated, that as the gentlemen who were expected to open the discussion, were not present, he was not yet prepared to bring the subject in a proper form before the Society. He would mention, however, that he had had six cases of rupture of the uterus in his practice, and would ask the indulgence of the Society in a brief recital of them.

The first case occurred many years since; the woman had had three children, and had been delivered with instruments previous to the present confinement. In the morning of her sickness the pains were light, and the *os* undilated; toward evening, however, the pains became very severe, and there were apparent symptoms of sinking; pulse 150, with cold, clammy perspiration, the head of the child having, in the mean time, receded. In consultation with Dr. Brown, turned and delivered. No post mortem could be obtained, but there was an apparent deformity of the sacrum, and the *rent* was in the posterior portion of the uterus.

In the second case, the woman had been previously delivered with instruments. When taken sick, in the morning, the pains were insignificant, and continued so throughout the day. In the evening, while sitting up, she suddenly sank back exhausted; she complained of no pain, but the pulse began to grow very quick and feeble, and symptoms of prostration were very apparent. On examination found no receding of the head, and no *two tumors* in the abdomen. In this emergency Dr. Brooks was called in, and we concluded that rupture had taken place; determined to turn and deliver as soon as possible, but the friends would allow nothing to be done. Was sent for again the next morning, but the patient died soon after his arrival. The post mortem revealed a rupture of the womb, which was not very extensive, and an ecchymosis and softening of the parts at the seat of the trouble.

The third case he saw in the morning, and as the pains were insignificant, he advised her to sit up, as labor might progress more rapidly. This, however, had no effect; the head began gradually to recede, slight hemorrhage occurred, and two tumors could be felt in the abdomen; the pulse was rapid and feeble, and symptoms of exhaustion set in. Turned and delivered immediately; enjoined perfect rest, and administered opiates. The next morning she said she felt better, but the pulse was more rapid and feeble, and she died in two or three days in a sort of *collapse.*

The fourth case was seen by Dr. Clark in the morning, when the pains were insignificant, and the *os* undilated. In the evening was sent for in great haste, and saw her with him. Detected rupture, and immediately turned and delivered. The woman died on the seventh day.

In the fifth case the pains were at first languid and labor tedious; after a while the head came down on the perinæum, and the pains became very severe. All pain, however, soon ceased; there was no hemorrhage, and no receding of the head; the pulse was rapid and feeble, and symptoms of prostration very evident. Having found, on his return with the instruments, that the patient was more prostrated, he determined to turn and deliver. She died the next day. On post mortem the *rent* was found in the posterior portion of the organ, at the junction of the cervix with the body.

The sixth case occurred recently, and was of great severity. The woman had been twice delivered with instruments in Ireland. On examination, found that the head had not engaged in the superior strait, and detected a deficiency in the diameter of the pelvis. Saw her several times during the day, but the pains were insignificant. In the evening symptoms of exhaustion set in, but on examination could detect no two tumors in the abdomen; the head had fallen forward on the pubic bone, but was fixed enough to perforate. Concluded, however, to turn and endeavor to deliver. The body was extracted without much difficulty, but the head could not be pulled down, the occiput being entirely out of reach. Gave chloroform and ether, but the pulse becoming more feeble and rapid, had to be very cautious in their administration. Having found it impossible to deliver the head, concluded to decapitate first, and afterwards extract the head. Thought at one time that she was dead, but she rallied again a little, and she was left alone with the head undelivered. Death occurred on the following day. No medicines were given in any of these cases, and the difficulty occurred when the pains were insignificant. What, he would ask, was the best mode of treatment in these cases? If gastrotomy were resorted to, we would only have a wound remaining, which would be no worse than the rupture itself. It was a very fatal disease, and he imagined that in the reported cases of recovery, there was merely a *slitting* of the cervix, and not a rupture. The use of anæsthetics was very prejudicial, as it caused too much depression of the nervous system; and he thought no physician was authorized in delivering a woman *in extremis* merely to have it to say that she did not die undelivered. The causes of rupture of the uterus, in his opinion, were from some change in the uterine walls, a fatty degeneration of portions of the muscular fibres, and thinness of structure, or pressure on the sharp edge of the promontory of the sacrum. In a majority of cases, he thought, this accident occurred where there was deformity of the pelvis, and that it usually occurred at the junction of the cervix with the body of the uterus. If the uterus were perfectly developed no rupture would ensue, no matter how severe the pains. None of the children were born alive.

Dr. Hart had never had a case of the kind in his practice, but had seen one in 1849, in a lady, in her eighth confinement, the patient of another physician. At the commencement of labor she complained of coldness of the extremities, and symptoms of exhaustion were very evident, although the pulse was natural, and the countenance good. Delivery was effected as soon as possible, but in an hour or two afterwards she complained of nausea, the pulse became rapid and feeble, and death soon ensued. Two weeks previously she had injured herself, and complained at the time of feeling something give way. The child was alive. The post mortem revealed clots of blood in the uterus, and a rupture at its neck. The statistics in regard to these cases could not be relied upon.

Dr. Byrne considered these cases of deep interest to every obstetrical practitioner, and though he might not be able to offer anything new on the subject, he still hoped that considerable information might be obtained. Some authors have entered largely upon the consideration of rupture of the uterus, while others have scarcely noticed it, merely advancing some common-place remarks, and adverting to statistics. So much discrepancy, however, exists among these, that no reliance can be placed upon them.

The Causes of Rupture of the Uterus he would divide into three classes—1st, Maternal; 2d, Fœtal; 3d, Accidental.

In the 1st class he would rank *deformities*, *alteration* and *softening* in the uterine tissues; *degeneration* produced by traumatic inflammation, or pathological changes. In this class might also be mentioned *excessive* and *irregular uterine contraction*, where degeneration of the muscular tissue exists. In the 2d class we have *abnormal* fœtal developments of the head, and mal-positions of the fœtus in utero. In the 3d class may be enumerated *unwarranted delay* in the application of the forceps or in rectifying mal-positions; and lastly the administration of ergot.

With regard to the frequency of rupture, no reliance can be placed upon statistics.

The location of this trouble, however, is situated, generally, in the anterior portion, at the junction of the cervix with the body. Saw the first case in Dublin in 1843. The woman was in her seventh confinement, and without any extreme uterine contractions, the symptoms of rupture set in. The skull was perforated, and delivery effected. The woman recovered.

The 2d case occurred in a stout, robust woman, the mother of four children, and residing in South Brooklyn. On examination found the head pressing on the perinæum, and expected delivery to take place at any moment. Suddenly, however, she screamed out, but complained of no pain; the face became pale, the hands white, and great distress evident; the extremities were cold, and in an hour she was almost pulseless. Delivered with the forceps, but she died in twenty-four hours afterwards. The rupture was situated in the anterior portion of the uterus.

The 3d case occurred in a woman, the mother of four children. The pains were very irregular and inefficient, and continued so for twenty-four hours, when he was again called upon. Found the woman prostrated, with feeble pulse, pale countenance, cold extremities, and cessation of all uterine pains. On examination no deformity could be detected.

Another case happened in a woman, whom he had previously attended, and whom he had at the time advised, if she again became pregnant, to have labor brought on at seven months. She went, however, her full time, and was sick for three days, when symptoms of rupture set in. In this case the accident occurred on the right side. The rest of the cases were similar to those already mentioned.

He did not think it necessary to dwell upon the symptoms of rupture; they were very plain and simple, and easily recognizable. There is an entire cessation of uterine action, great prostration, and almost invariably, *coffee ground* vomiting.

The treatment of these cases has excited much controversy, both at home and abroad.

When the pelvis is so deformed that it is morally certain the head cannot pass through, then the treatment should begin at once, and no delay should occur. If the rupture had already taken place, he would perform gastrotomy. He referred to the much talked of case of Elizabeth Sherwood, and thought it *fabulous;* for he could not see how it was possible for a child's head to be drawn through a space of $1\frac{3}{4}$ inches, nor how the woman could survive the operation. If there is rupture, gastrotomy will not make the woman worse, and he considered it, therefore, the proper treatment. But he could not see any reason why a woman should be delivered *in extremis,* merely to have it to say that she did not die undelivered.

With regard to the *after treatment* of these cases, no reliance could be placed upon statistics. In thirty-four cases, there were two recoveries. One of these was very peculiar, recovery having taken place under the calomel and jalap treatment. Our only resort, however, seems to be in opiates, and in keeping the vagina syringed with warm water. When recovery occurs in these cases, rupture is apt to recur at the next labor. Professor Simpson has clearly proved that the head of the male child, at full development, is larger than that of the female.

Dr. Housel felt very much interested in this subject. He had seen one of the cases mentioned by Dr. Byrne, where labor had progressed for thirty hours before rupture occurred. He believed that recovery has taken place after this accident, and the statistics would prove it.

Dr. Hawley read a report of two cases, where the two women had all the characteristic symptoms of ruptured uterus, and recovery had taken place.

Dr. Conkling thought that there might be considerable doubt whether there was, in reality, rupture of the uterus in the cases reported by Dr. Hawley.

Dr. Chapman remarked that there should be some *positive* symptoms before a case of the kind could be reported as having recovered. A mere hemorrhage, or severe pain in the epigastric region, could not be considered diagnostic. The detection of *two tumors* in the abdomen was, in his opinion, the only positive sign of the accident. If the *rent* was large and the child had passed into the peritoneal sac, he doubted whether recovery could take place.

---

### REGULAR MEETING, JUNE, 1864.

### *Cancer.*

Dr. Hutchison presented a specimen of cancerous disease in the two mammary glands. In the right breast the trouble commenced about six months previous to its attacking the left side. The tumor gradually increased in size, and was hard and nodulated, with retraction of the nipple. The patient complained of no severe pain, except after violent exercise, and by her own request, the diseased mass was removed. The right breast was easily removed; no hemorrhage occurred, but suppuration was profuse. The left healed by the first intention. The disease was carci nomatous in character. Could not trace any hereditary taint.

The second specimen was of cancer of the right breast, of three years' standing. The tumor was not large, but hard and moveable, and closely adherent to the top of the breast. There was no retraction of the nipple, and the patient complained of no severe pain. The operation was performed three weeks ago, and when first removed, the gland looked like a mass of fat, but now the diseased tissues can be distinctly felt. It was found to be of the encephaloid variety, which is of very rare occurrence in the mammary gland.

Dr. Hutchison thought an operation in these cases was proper, although it might not prolong life. He did not know of any case where the patient lived more than four years. Dr. Buck, however, mentioned a case where the patient lived for twenty years.

Dr. Enos stated that when in London in 1858, he saw a cancerous tumor removed from the breast of a lady, who, according to report, had been operated upon for the same trouble, twenty years previously. The patient enjoyed pretty fair health.

Dr. Hutchison remarked that Dr. Watson had a case of cancer of the genitals, where a cancerous mammary gland had been removed twenty years previously.

*Cystic Tumor.*

Dr. Hutchison also presented a cystic tumor, removed from the right leg of a lady. Had punctured it some time ago, and the fluid ran out; but the trouble soon after returned, and the tumor was removed on the 17th instant. Thought the only treatment in these cases was, to dissect the *sac* out *entire.*

*Catalepsy—Diabetis Millitis.*

Dr. Cullen reported the occurrence of two comparatively rare cases of disease in one family. One a case of catalepsy, and the other diabetis millitis; the latter was of especial interest on account of the excessive secretion—from three to twelve quarts of urine in twenty-four hours. S. g. '34—'40, and syrupy; the per cent. of sugar not ascertained. The patient is a female, aged 12 years; keeps her flesh pretty well, with tolerable appetite, and diminishing thirst, which has been excessive. The tongue is *beefy*, but not dry.

Dr. Catlin is disposed to regard the secretion of sugar as being due to some abnormal state, or irritation of some portion of the pneumogastric nerve, in accordance with certain experiments of Dr. Hunt, jr., in 1860, and that the sugar may be manufactured by the liver, kidneys or lungs.

Dr. Enos cited the writings of certain French pathologists—Faver and Bernard—who maintain that the secretion of sugar under such experiments only takes place during the act of dying. The treatment of the case has been chiefly dietetical—avoidance of starchy and saccharine matter. Has given bicarbonate of soda, but doubts its benefits. Paliation merely is all he anticipates; knows of no curative means.

### *Cystitis.*

Dr. Catlin reported a case of Cystitis (?) The gentleman called yesterday, presenting a ʒij phial of urine, with half an inch depth of deposit. On microscopical examination found it to contain pus, and crystals of oxalate of lime—square. The question arises, whence the pus? Patient complains of pain in lumbar region. The urine gelatinizes with potash. Nitric acid slightly thickens it, but heat clears it up again.

Dr. Johnson regarded it as a case of cystitis, there being no evidence of renal disease. In evidence of which he cited a similar case. There having been in this case no complaint calling attention to bladder. Dr. J. did not know of any other microscopic characteristics than pus. The case finally resulted in rapid failure and death.

Dr. Bell cited two cases in the City Hospital. One of them evidently improving under the injection of nitrate of silver 10 grs. to the ℥i—½ used at a time. A weaker solution has not been followed by beneficial results.

Dr. Cullen cited a case complicated with prioprism and nocturnal seminal emissions. He has not been able to relieve the patient, and has recently advised him a sea-voyage. Dr. Cullin's experience in such cases, generally, especially if they have existed long, is unfavorable to recovery.

### *Caries.*

Dr. Johnson reported a case and showed specimen of amputation of leg for caries of lower third of tibia. The case having been before trephined. But the disease progressed, and finally became so extensive as to require amputation, which was performed yesterday. The patient was a female, aged 42, and Dr. J. thinks of probable syphilitic cachexia. Otherwise he is unable to account for the persistent progress of the disease, in spite of frequent removals of all diseased bone. On discussion, the general expression of opinion was to the effect, that the caries was the result of periostitis, and to such a degree as to have destroyed the nutrition of the bone.

---

### REGULAR MEETING, SEPTEMBER, 1864.

### *Hernia.*

Dr. Enos presented a specimen of umbilical hernia, removed from a man 56 years of age, short and fleshy, weighing 280 pounds. He had suffered from it for a number of years, and wore a truss, but the protrusion never fully returned. Several weeks since more of the contents were forced through the opening, and he was immediately seized with pain; but the wife, thinking it merely a colic, applied mustard to his bowels. Two days afterwards he was seen by his physician, when collapse was apparent—a weak and rapid pulse, body bathed in perspiration, and vomiting of greenish matter.

On examination the *tumor* was found to be twenty inches in circumference, and evidently strangulated. Anæsthetics were immediately administered, and the parts cut down upon, the peritoneal sac being opened during the operation. The omentum was found to have protruded, and had formed a kind of sac on the abdominal wall. The adhesions were broken up, and the contents returned into the abdomen. The man gradually sank, and died in forty-eight hours afterwards.

On post mortem, a portion of the small intestines (probably the ileum) had also been found to have protruded, and this had produced the grave symptoms.

Doctor E. also presented a tumor, which he had removed from the breast of a healthy, robust girl. She had noticed it for two or three years, its growth being very slow. At first it gave her no trouble, but after a while she complained of a pain shooting around the shoulder. About a week ago she came to his office, and on examination found in the right breast a *tumor*, quite moveable. Neither the integuments nor the glands in the axilla were involved; the health was good, with the exception of some irregularity about the menses. Found upon inquiry, that some years previously, some of the members of the family had died of cancer. Advised its removal, and the tumor was taken out, the parts healing up kindly. The tumor was modulated, the structure being somewhat like the gland itself, and only attached to the breast by the tendinous processes. This complaint is called by Sir Astley Cooper, the *chronic mammary tumor*, or *nodulated tumor*. It will sometimes disappear, and if not painful, would advise the "let alone" treatment. Under the microscope it was found to consist of a fibrous structure, attached to the glandular substance.

Dr. Hart stated that about thirty years ago he attended a lady in her confinement who, after the secretion of milk was well established, complained of a pain and swelling in the right breast. Examined and found a tumor, resembling the one described by Dr. Enos. The lady remarked that she had noticed a little lump there for a long time, but it did not give her any trouble. In this instance suppuration took place, and the tumor entirely disappeared.

Dr. Wm. Gilfillan desired to know, what was the *efficient cause* of death in the case of umbilical hernia?

Dr. Enos thought it was from *exhaustion*. The patient seemed to recover completely from the anæsthetic influence, but there was a gradual sinking after the operation. The peritonitis was not extensive. The trouble was also aggravated by the cathartic medicines he had taken. He was, also, very fat, and with little recuperative energy, and when first seen he had been sick for several days, was very weak, and had been vomiting a great deal.

Dr. Wm. Gilfillan thought it a very instructive and very interesting case. This summer, while in the country, he was called to see an elderly lady, who was attacked with all the usual symptoms of cholera morbus, and was treated for such. The complaint not subsiding, a further examination was made, and a small, moveable tumor found in the femoral region. Concluded there was a hernia, and two hours afterwards operated. While the chloroform was being administered, she vomited some stercoraceous matter. The woman recovered. Upon inquiry found that the tumor had been coming and going for the last two or three years, but as it did not give her any trouble she did not notice it. Mentioned this as an instructive case.

Dr. Enos stated that a short time ago he saw an old lady, who had heen suffering for years from an *irreducible femoral hernia.* She was suddenly seized with vomiting and excessive pain, and on examination found a large tumor in the groin. In cutting down upon the parts found the omentum, which is not a common occurrence in this region. A portion of the intestines was also forced out, which, becoming constricted, produced the alarming symptoms. About 2½ inches of omentum were forced out, the pedicle being 1½ inches in diameter. When the omentum is adherent in such cases, he thought it just as safe to cut it off.

### *Ileus.*

Dr. Landon related a case of a man who was taken ill in February last, with violent colic, followed by obstinate hiccough and coughing for several weeks. In March discovered a small tumor in the iliac fossa, which gradually increased in size, and was slightly tender. It continued to increase until August, when fluctuation was discovered, which extended towards the crest of the ilium. A large abscess (on the side) was evident, which was opened, discharging at first blood and serum, followed by an oily, fetid fluid, and afterwards by healthy pus. A week previous to death a fœcal discharge occurred. With the appearance of the tumor there was *constipation* for two or three days; during the time of the discharge the bowels were *regular;* when the fœcal discharge occurred *diarrhœa* set it. The abscess on the side was immense, and occupied a large portion of the iliac fossa, passing up and over the crest of the ilium, and denuding the bone for the space of an inch. The head of the colon, and the bowels adjacent, were permanently attached to the ilium; the extremity of the *appendix vermiformis,*

as may be seen in the specimen, was closed in under the head of the colon. The abscess commenced with the ascending colon, and extended down the iliac fossa. The swelling was just inside of the anterior superior spinous process.

Dr. Enos remarked that the trouble in this case commenced, most likely, in the appendix vermiformis. Some fœcal matter might have collected there, giving rise to inflammation and ulceration, and finally peritonitis. The peritonæum may have become adherent, and the matter thus found its way through that membrane, and over the crest of the ilium. This, of course, is an unusual occurrence, as penetration of the appendix produces violent iuflammation; still, he thought the inflammation commenced in that part of the intestine.

Dr. Wm. Gilfillan did not agree with Dr. Enos in regard to the origin of the trouble. He had examined the specimen very carefully, and thought the symptoms would indicate that the disease was caused by some obstruction at the *ileo cœcal* valve, producing inflammation and ulceration into the surrounding cellular tissue, the *abscess* finally extending over the crest of the ilium. Did not think that it could have commenced in the *appendix vermiforma.* Had often seen violent colic produced by eating apples, the inflammation being kept in bounds only by large doses of opium.

Dr. Enos remarked that upon a more careful examination of the specimen, he agreed with Dr. Gilfillan, that the disease had commenced at the ileo-cœcal valve.

